# Microarray-Based Allergy Diagnosis: Quo Vadis?

**DOI:** 10.3389/fimmu.2020.594978

**Published:** 2021-02-12

**Authors:** Huey-Jy Huang, Raffaela Campana, Oluwatoyin Akinfenwa, Mirela Curin, Eszter Sarzsinszky, Antonina Karsonova, Ksenja Riabova, Alexander Karaulov, Katarzyna Niespodziana, Olga Elisyutina, Elena Fedenko, Alla Litovkina, Evgenii Smolnikov, Musa Khaitov, Susanne Vrtala, Thomas Schlederer, Rudolf Valenta

**Affiliations:** ^1^Division of Immunopathology, Department of Pathophysiology and Allergy Research, Center for Pathophysiology, Infectiology and Immunology, Medical University of Vienna, Vienna, Austria; ^2^Laboratory of Immunopathology, Department of Clinical Immunology and Allergology, Sechenov First Moscow State Medical University, Moscow, Russia; ^3^Department of Allergology and Clinical Immunology, NRC Institute of Immunology FMBA of Russia, Moscow, Russia; ^4^Karl Landsteiner University of Health Sciences, Krems, Austria

**Keywords:** allergy, allergen, IgE, molecular diagnosis, microarrayed allergens, allergen chip, precision medicine

## Abstract

More than 30% of the world population suffers from allergy. Allergic individuals are characterized by the production of immunoglobulin E (IgE) antibodies against innocuous environmental allergens. Upon allergen recognition IgE mediates allergen-specific immediate and late-phase allergic inflammation in different organs. The identification of the disease-causing allergens by demonstrating the presence of allergen-specific IgE is the key to precision medicine in allergy because it allows tailoring different forms of prevention and treatment according to the sensitization profiles of individual allergic patients. More than 30 years ago molecular cloning started to accelerate the identification of the disease-causing allergen molecules and enabled their production as recombinant molecules. Based on recombinant allergen molecules, molecular allergy diagnosis was introduced into clinical practice and allowed dissecting the molecular sensitization profiles of allergic patients. In 2002 it was demonstrated that microarray technology allows assembling large numbers of allergen molecules on chips for the rapid serological testing of IgE sensitizations with small volumes of serum. Since then microarrayed allergens have revolutionized research and diagnosis in allergy, but several unmet needs remain. Here we show that detection of IgE- and IgG-reactivity to a panel of respiratory allergens microarrayed onto silicon elements is more sensitive than glass-based chips. We discuss the advantages of silicon-based allergen microarrays and how this technology will allow addressing hitherto unmet needs in microarray-based allergy diagnosis. Importantly, it described how the assembly of silicon microarray elements may create different microarray formats for suiting different diagnostic applications such as quick testing of single patients, medium scale testing and fully automated large scale testing.

## Background

The major difference between allergic patients and healthy, non-allergic subjects is that allergic patients produce IgE antibodies against certain environmental antigens, termed allergens, whereas non-allergic subjects produce IgG antibodies (1, 2). IgE antibodies bind specifically to high (Fc*ε*RI) and low affinity receptors (Fc*ε*RII) for IgE present on inflammatory cells which become activated by IgE-allergen immune complexes to release inflammatory mediators, cytokines, and proteases and/or to activate allergen-specific T cells (3, 4). Therefore, allergen contact induces in allergic patients containing specific IgE, allergic inflammation in different tissues and organs leading to a variety of allergic symptoms comprising rhinoconjunctivitis (hay fever), asthma, skin inflammation, gastrointestinal symptoms, and systemic symptoms such as anaphylactic shock (5). By contrast, IgG recognition of allergens does not trigger allergic inflammation because allergen-IgG immune complexes cannot bind to Fc*ε* receptors and thus fail to trigger allergic inflammation. IgE antibodies occur in very small concentrations in the blood and therefore were identified only in 1966 (6). Due to their importance for triggering allergic reactions already in 1967 the first serological test for measuring allergen-specific IgE in the blood of allergic patients was developed and termed radioallergosorbent test (RAST) (7). Before the discovery of IgE antibodies, allergic sensitization was diagnosed by exposing subjects with suspected allergic sensitization to extracts made from the disease-causing allergen sources in order to study if this would induce immediate allergic inflammation. One of the first descriptions of controlled allergen provocation dates back to a study performed by Charles Blackley in 1873 (8). Since the induction of allergic inflammation resulting from the activation of mast cells by IgE-allergen immune complexes occurs within few minutes, IgE-associated allergy was also termed immediate type hypersensitivity in the classical description of the four types of immunological hypersensitivity of the immune system published by Coombs & Gell (9). Accordingly the diagnosis of allergy has been based on three elements, one is the case history trying to relate the occurrence of allergic symptoms in a patient to exposure to certain allergen sources; the second element is trying to induce allergic reactions in the patient by exposing the person to the allergen source and recording of subsequent allergic symptoms; and the third by confirming IgE sensitization by demonstrating the presence of IgE antibodies specific for the allergen source in the blood or tissue fluids of the patient (10). Traditionally, testing is performed exactly in the described order by starting with the anamnesis followed by provocation testing and final confirmation of sensitization by measuring specific IgE antibodies.

## Traditional Forms of Allergy Diagnosis

Traditional allergy diagnosis always starts with a detailed anamnesis trying to identify the presence or absence of allergic symptoms. The next step is to try associating the occurrence of symptoms with contact to certain allergen sources and to verify that controlled exposure to allergen extracts prepared from the allergen source will elicit an allergic reaction. For this purpose, allergen extracts are prepared from the natural allergen sources. These allergen extracts represent mixtures of allergenic and non-allergenic, potentially also irritating substances which may elicit an inflammatory reaction without underlying IgE sensitization. Some examples are the presence of histamine in fish or adverse reactions to milk due to lactose intolerance (11). Accordingly the next step for confirming the condition of an IgE-associated allergy is to verify that the patient serum contains IgE antibodies which react specifically with the allergen extract. However, the demonstration of the presence of allergen-specific IgE with allergen extracts is problematic. First of all, the disease-causing allergen molecules cannot be identified with allergen extracts because they represent mixtures of different allergen molecules and non-allergenic materials. Furthermore, the quality of allergen extracts may strongly vary and depend on various factors which are out of the control of the manufacturer. For example, certain allergens may be lacking in certain extracts (12) and there may be contaminations with allergens from other sources just to name a few problems. It also seems that allergen extracts for *in vivo* provocation testing are becoming less available because they do not meet current standards for medical products (13). Accordingly the use of allergen extracts has several disadvantages which are reviewed in (14). In order to address the urgent needs and bottlenecks of diagnostic allergens the European Academy of Allergy and Clinical Immunology (EAACI) has founded a task force which has set up an action plan to maintain the supply of diagnostic allergen extracts (15). This action plan comprises i.) a simplification of authorization, ii.) specific regulations for special types of extracts, iii.) new models beyond the current model of homologous allergens, iv.) simplification of pharmacovigilance reporting, v.) reduction of regulation fees and vi.) reimbursement for diagnostic allergen extracts. Nevertheless, the practice of traditional allergy diagnosis becomes now challenged with the appearance of molecular allergy diagnosis.

## Molecular Allergy Diagnosis

Shortly after the first allergen-encoding DNAs had been isolated (16–18), the first two studies were published showing that one can replace complex allergen extracts such as birch pollen or grass pollen extract with a few defined recombinant allergen molecules for IgE-based serological diagnosis without losing sensitivity or specificity (19, 20). These results were quite surprising because at that time it was thought that allergen molecules may exist in different isoforms with variable IgE reactivity and that it may be impossible to find one isoform which would be suitable for the diagnosis in all patients (21, 22). Furthermore, it was not clear if a few allergen molecules would be sufficient to cover the IgE epitopes of whole allergen extracts. The early studies performed with recombinant allergens for diagnosis also indicated that patients who are sensitized to a certain allergen source may react with different molecules in this source and accordingly show different clinical phenotypes that need “patient-tailored treatment concepts” (19). Subsequently, an increasing number of allergen molecules from different sources were produced and recombinant allergens became available for the first time in the most commonly used fully automated *in vitro* allergy diagnosis system, the ImmunoCAP system (23). However, the principle of ImmunoCAP testing was that one test provided only one information so that for each allergen source several allergen molecule-based tests would be needed to cover the spectrum of the allergens of the allergen source. Thus molecular testing with single allergen molecules would have increased the costs for testing considerably. It was therefore, clear that using this test one would always start testing for allergen-specific IgE with allergen extracts and only if deemed necessary and affordable, one would continue with molecular testing.

In order to utilize the increasing numbers of allergen molecules which were developed by time for diagnostic testing, new test platforms were needed. It was a co-incidence that microarray technology became available for printing nucleic acids onto chips leading at that time. The first DNA chips were manufactured by the company Affymetrix which was based in Santa Clara, California, and a similar technology was applied by the Vienna start-up company VBC Genomics headed by Manfred Müller from Vienna, Austria which had the instruments for printing microarrays. In a collaboration between Manfred Müller and Rudolf Valenta, which was the first to develop chips containing microarrayed allergen molecules were then developed (24). which were not only one of the first protein arrays for diagnostic purposes but also represented the first microarrays for *in vitro* allergy diagnosis (24). These micro-arrays contained more than 90 different allergen molecules from different allergen sources provided by researchers from all over the world. The exciting thing with allergen microarrays was that one could test IgE reactivity simultaneously to a large number of different allergen molecules with a few microliters of serum or other body fluids. Compared to other existing allergy test systems the allergen chip thus represented a breakthrough which may be also considered a “disruptive technology” because it had the potential to change allergy diagnosis completely. One year after the appearance of the study describing the first allergen chip, the concept of microarray-aided allergy diagnosis was considered the first time (25).

**Figure 1:**
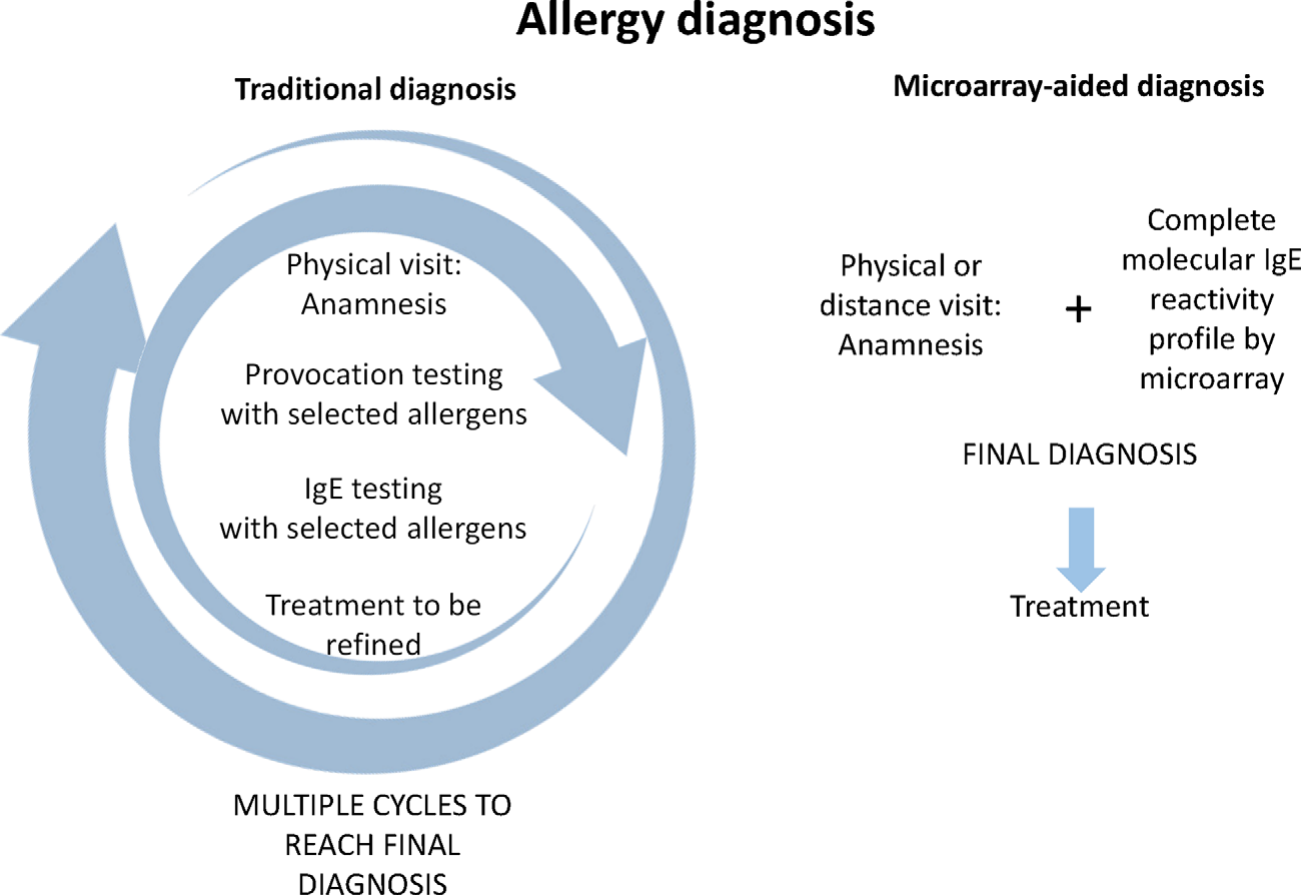
Comparison of traditional allergy diagnosis and microarray-aided allergy diagnosis. In traditional allergy diagnosis (left part) an anamnesis of allergic symptoms is recorded which serves as the basis for targeted provocation testing, usually skin testing with a limited number of allergen extracts selected according to the anamnesis and eventually collection of a serum sample for measuring IgE specific for the suspected allergen sources. In the best case, the patient receives first treatment suggestions according to skin test results. Usually at least one, but often several additional visits are necessary to adjust the treatment to the IgE test results and/or to conduct further targeted testing to determine more precisely the patients sensitization profile and to further adjust treatment. Regarding microarray-aided allergy diagnosis (right part), it can be envisioned that the first visit can be conducted even in a virtual, telemedicine-like form because no provocation testing is required. The anamnesis and complete molecular IgE reactivity profile would be available to the specialist online who could then prescribe precise treatment taking clinical information and the complete sensitization profile into consideration.

**Figure 1** provides a comparison of traditional allergy diagnosis, which in principle is an approach driven by the hypothesis developed by the physician based on the anamnesis. According to the information collected by the anamnesis the physician selects certain allergen sources for a first round of *in vivo* provocation testing which is usually conducted by skin testing, and eventually the confirmation of IgE sensitization by serology is performed. According to the *in vivo* provocation results which can be evaluated during the first visit, the physician may already make first preliminary treatment recommendations. Results from serology are usually not available on the same day, and often it may turn out that the original hypothesis, what the culprit allergens might have been, may need refinement through additional anamnesis and repeated testing (**Figure 1**, left). Therefore traditional allergy diagnosis requires usually multiple visits until a more or less complete picture of the sensitization profile of the patient has been obtained. It is needless to say that the traditional allergy testing path may become challenging because it may require repeated consultations and thus long time to reach a final diagnosis, and this may reduce patients’ compliance (**Figure 1**).

By contrast, microarray-aided allergy diagnosis has the potential to reduce the time to reach the final diagnosis and to select the correct treatment albeit it may be initially more expensive because it would place the assessment of the complete sensitization profile of the patient already at the beginning of the first consultation. One could imagine that based on validated allergy questionnaires such as the ISAAC questionnaire (26) already the general practitioner (GP) initiates microarray-based allergy screening in subjects with suspected allergy. The patient could then have a first consultation with a specialist presenting already the anamnesis and the complete molecular IgE reactivity profile so that the specialist can immediately determine the best treatment practice. In contrast to the traditional “hypothesis-driven approach”, this pathway is reminiscent of a “discovery approach” because the comprehensive IgE test result might stimulate the specialist to refine the anamnesis according to the IgE sensitization profile. Obviously the microarray-aided diagnosis would need *in vivo* provocation testing only in certain cases for confirmatory purposes, if at all and hence the consultation could be performed also in a telemedicine-based format. The advantage of such a telemedicine-based approach is that it reduces the number of visits, avoids eventual long travelling, and remote areas in a country can easily benefit from specialist knowledge (**Figure 1**, right). In this context the possibilities of mobile health (mHealth) technology using mobile communication devices to support and improve health-related services, data flow, patient management, surveillance, and disease management should be mentioned. A task force of EAACI has reviewed all these possibilities, discussed advantages, limitations, and risks such as data protection, and provided recommendations (27). In the context of precision medicine, the potential of changing the practice from clinician- to patient-centered health care is highlighted.

Some recent reports underline the potential of molecular diagnosis in the field of health economics and suggest that it can help to save costs for the diagnosis of respiratory allergies (28) and food allergies (29).

Currently, allergy diagnosis is still dominated by the traditional approach of diagnosis as indicated in **Figure 1**, but more and more specialists in allergy start to use molecular allergy diagnosis, and already quite a few prefer microarray-aided diagnosis as shown also in **Figure 1**. There are many different reasons for the different preferences. For example, traditional allergy diagnosis is often preferred because it is currently reimbursed by the health care system, whereas serological diagnosis and, in particular microarray diagnosis, requires laboratory facilities and a different mode of reimbursement shared between specialists and diagnostic laboratories. Factors limiting the increased use of microarray-guided diagnosis are that practitioners must be skilled in interpreting complex molecular test results and/or have well-trained algorithms for clinical decision making (*i.e.*, clinical decision support systems) available. Information on sensitizations not linked to symptoms require appropriate and time-consuming information of patients, and certain allergen molecules for obtaining complete results may still need to be discovered and included in the micro-arrays. Finally, it will be necessary to reduce the costs of micro-arrays, which are often driven not only by costs of goods for manufacturing but also by costs due to quality control, validation, and registration. However, once the latter issues are resolved one may expect that microarray-based allergy diagnosis will become a highly cost-effective diagnostic tool. One must consider that microarray-based allergy diagnostic approaches can provide more than hundred individual test results from one sample and present the comprehensive picture of allergen sensitization, which can be used for precision medicine treatment. In fact, unrecognized and untreated allergy is a major cost factor for the management of allergic diseases. With the adherence to proper treatment and the precise diagnosis and proper management of allergic diseases, it has been estimated that high costs can be saved (30, 31).

## Measurement of Allergen-Specific IgE and IgG Responses With Microarrayed Allergens

Microarrayed allergens can be used for measuring simultaneously IgE reactivity to a large number of different allergen molecules with very small volumes of serum. In this context, it should be mentioned that it has been shown that allergen-specific IgE can be also measured in plasma and other body fluids such as nasal secretions and in milk samples (32–35). Moreover, it has been shown that dried blood spots on paper can be recovered for specific IgE and IgG measurements which allows sending serum samples as paper dried blood spots in simple envelops without requiring expensive packaging, cooling, and sophisticated transportation (36). Several recent studies have confirmed the importance of allergen-specific IgG antibodies for the protective effects of AIT, and the measurement of allergen-specific IgG antibodies which block allergic patients IgE binding to allergens is therefore considered as an important biomarker for the clinical efficacy of AIT (37). Accordingly, certain commercial allergen arrays allow measuring specific IgE and IgG antibodies (*e.g.* Thermo Fisher ImmunoCAP ISAC Immuno-solid-phase Allergen Chip, which contains 112 allergens from 51 allergen sources) (38); however, this has not been shown for all available allergen arrays (*e.g.*, MADx Allergen Explorer ALEX; containing 282 allergens: 156 extracts and 126 components) (39). The measurement of allergen-specific IgG antibodies in cohorts has provided new insights in beneficial functions of IgG. For example it has been suggested that allergen-specific IgG transmitted from the mother to the child *via* the placenta during pregnancy may protect the off-spring from allergic sensitization (40). Likewise, evidence has been provided that the production of allergen-specific IgG antibodies follows different pathways and mechanisms than those involved in the production of allergen-specific IgE antibodies (2, 41–43).

## Examples for the Use of Microarrayed Allergens in Allergy Research

Microarrayed allergens have been used in research to address several questions. For example, it has been shown that adult allergic patients do not change their IgE sensitization profiles for a decade demonstrating that there is no acquisition or loss of IgE sensitizations in adult allergic patients (44). Furthermore, information was obtained about the characteristics of IgE sensitization profiles in different populations. For example, it was found that sensitization to clinically relevant grass pollen allergens is rare in tropical climates and that most of the grass pollen-specific IgE is directed to non-allergenic carbohydrate epitopes (45). In another study it was observed that man-made changes of the environment as for example obtained by replanting of certain plants can alter the allergic sensitization profiles towards plant-derived allergens in populations within two generations (46).

However, without any doubt, the most exciting results were obtained when microarrayed allergen molecules were used to study the development of the allergic sensitization profiles in longitudinal birth cohorts allowing to analyze the evolution of IgE sensitization profiles from birth to early adolescence (47, 48). Importantly it was found that allergy frequently starts with early asymptomatic IgE sensitization and that early assessment of IgE sensitization profiles and IgE-levels allow predicting the development of allergy later in life (49–52). This finding suggests that for allergy similar as for other diseases such as cancer, cardiovascular diseases, and metabolic disease, early screening in the form of a preventive medical examination by determination of IgE sensitization profiles early in life might allow initiating preventive measures (*e.g.*, allergen avoidance, early allergen-specific immunotherapy) (53–55) to prevent the development of allergic symptoms (*i.e.*, secondary prevention) later in life.

## Precision Medicine by Chip-Based Allergy Diagnosis

In allergy, like in other important diseases, it has become clear that it is necessary to transform healthcare towards the principles of “P4 Medicine” for predictive, preventive, personalized (precision), and participatory medicine by developing new diagnostic and predictive tests as well as therapies and preventive strategies which affect the course of disease or prevent the development of disease (56). Allergy is ideally suitable for a precision medicine approach because patients are sensitized to different allergens and allergen combinations and suffer from a wide variety of symptoms which may change during the course of disease. Furthermore, there are several different strategies for the treatment of allergy available which require the identification of the disease allergens. It is also clear that early preventive measures should be more effective than late mending of severe disease (5, 57). Accordingly, it has been suggested that molecular allergy diagnosis improves treatment especially in pediatric allergy (58). In this context, examples of how molecular diagnosis helped in the diagnosis of children suffering from complex allergic sensitizations and tailoring the treatment according to the needs of the children should be mentioned (59). Furthermore, evidence accumulates that allergic phenotypes and symptoms are associated with certain patterns and/or levels of allergen-specific IgE in children and adult allergic patients suggesting that serological surrogate parameters for diagnosis can be developed (60–63).

### Microarrayed Allergens in Food and Respiratory Allergy

The diagnosis of food allergy is often challenging because the frequency of IgE-associated food allergy is often considered higher than it is in reality. For example, adverse reactions to cow’s milk due to lactose intolerance are much more common than IgE-mediated allergy (11). Although many clinicians consider only the double-blind, placebo-controlled food challenge as gold standard for the diagnosis of food allergy, this test is cumbersome, and there may be severe and even life-threatening side effects. Accordingly, alternative diagnostic tests are needed. So far a considerable number of food allergen molecules have been identified which are associated with severe, mild, or even no reactions allowing for serological testing of food allergy also by microarray-based IgE testing (64).

In this context a study demonstrating different IgE sensitization profiles in children suffering from severe peanut allergy and in peanut-sensitized but asymptomatic children should be mentioned (65). Screening for IgE sensitizations using a large panel of food allergen molecules is useful for several reasons. First, it allows testing simultaneously for IgE sensitization to a large panel of allergen molecules with high anaphylactic capacity to predict the risk of food allergy, and thus it may help to reduce hazardous food challenge testing (29, 66, 67). Second, and importantly, negative test results to a large panel of food allergen molecules are helpful in searching for other reasons of food intolerance beyond IgE-mediated allergy. Besides IgE testing to food allergen molecules, it has turned out that measuring IgE sensitizations to food allergen-derived peptides may be useful to discriminate patients with no or mild symptoms from those suffering from severe symptoms (68, 69). Microarrayed peanut allergen molecules were also used to investigate the course of peanut sensitization in childhood and to predict symptomatic peanut allergy in a birth cohort (70). Very recently it was found that oral allergy syndrome (OAS) to Bet v 1-related food allergen molecules of the pathogen-related protein 10 (PR10) family was associated with the levels of Bet v 1-specific IgE and the numbers of recognized PR10 molecules (61).

Microarrayed allergen molecules are not only useful for the diagnosis of IgE-mediated food allergies but are also considered for the diagnosis of asthma triggered by allergens in sensitized allergic patients (71). The two major trigger factors for asthma are allergens for patients with IgE sensitizations and infections with respiratory viruses, in particular with rhinoviruses (RVs) (72). For example, it has been shown for house dust mite allergy that children suffering from allergic asthma differ regarding their IgE reactivity profiles and ability to produce allergen-specific IgG antibodies (60). Children with asthma showed higher IgE levels to certain allergens, reacted with a larger number of molecules, and produced less allergen-specific IgG as compared to children suffering only from rhinitis (60). Furthermore, machine learning approaches have been suggested to identify pairwise interactions of IgE antibodies and their association with asthma based on microarray results (73). Allergen molecules from cat and dog, which are important for the development of respiratory allergy in childhood, have been identified in the Swedish BAMSE birth cohort (74).

For the diagnosis of RV-induced asthma, a chip containing peptides derived from the N-terminus of VP1 proteins from a representative number of RV strains covering RV-A, RV-B and RV-C species has been produced (75). This chip allowed measuring species-specific increases of RV-specific IgG antibodies in children who had experienced RV-induced asthma exacerbations, and cumulative IgG responses were higher in children with RV-induced exacerbations of respiratory illnesses (76, 77). Accordingly, it has been proposed to use microarrayed allergens and respiratory virus-derived peptides for diagnosis of allergen and/or RV-induced asthma and personalized treatment according to the test results (77).

### Microarrayed Allergens for Prescription and Monitoring of Allergen-Specific AIT

AIT is an allergen-specific form of therapeutic vaccination which is based on the administration of the disease-causing allergens or modifications thereof with the goal to induce allergen-specific protective IgG antibodies and alterations of the cellular immune response to reduce symptoms of allergy upon allergen contact. Accordingly, the accurate prescription of AIT requires that the culprit allergens are identified. Since allergen sources often contain cross-reactive allergens the identification of the culprit allergen source can be challenging. It has therefore been suggested to use marker allergen molecules which are specific for given allergen sources as diagnostic marker allergens for improving the prescription of AIT (78). The marker allergen concept can be applied to almost all allergen sources and accordingly has been suggested for several common respiratory allergen sources (79–81). The use of marker allergen molecules was suggested not only for refining the prescription of AIT but also for the monitoring of treatment response by measuring the development of allergen-specific IgG antibodies which are considered as biomarkers for the success of AIT (37, 82). In this context an interesting discovery was made which suggested that the ImmunoCAP ISAC microarray platform which utilizes small amounts of immobilized allergens is very useful for AIT monitoring. In fact, when small amounts of allergens are immobilized on the solid phase of immunological tests for detecting allergen-specific IgE, IgG antibodies can compete with IgE for allergen binding when they block epitopes recognized by patients IgE (83, 84).

When patients develop such blocking IgG antibodies in the course of AIT, these IgG antibodies will then compete with IgE antibodies which then causes a reduction of IgE binding similar as it is observed by skin testing when allergen-specific IgG antibodies block IgE-mediated mast cell activation thus leading to a reduction of skin responses. The reduction of allergen-specific IgE binding by blocking IgG antibodies can only be measured with IgE binding assays containing small amounts of immobilized allergens such as the ImmunoCAP ISAC chip but not by the traditional ImmunoCAP test which contains larger amounts of immobilized allergens (83–85). No other diagnostic platform with similar properties has been identified so far. In fact, two independent studies have shown the reduction of IgE binding to allergens by AIT-induced IgG antibodies on the ImmunoCAP ISAC platform and suggested it as a possible biomarker for AIT (86, 87).

Several studies support the concept of using molecular testing for the refined prescription of AIT (88), and the cost-effectiveness of molecular diagnosis as compared to traditional allergy diagnosis has been shown (89). Two more recent studies should be mentioned which have shown that it may be possible to enhance the success of AIT by selection of patients whose IgE reactivity profiles match the immunogenic components present in the AIT vaccines (90, 91). Accordingly, microarray-based molecular diagnosis seems to be well suited as companion diagnostic tool for the selection of patients for AIT and for the monitoring of treatment success.

## Unmet Needs in MicroArray Technology

**Figure 2:**
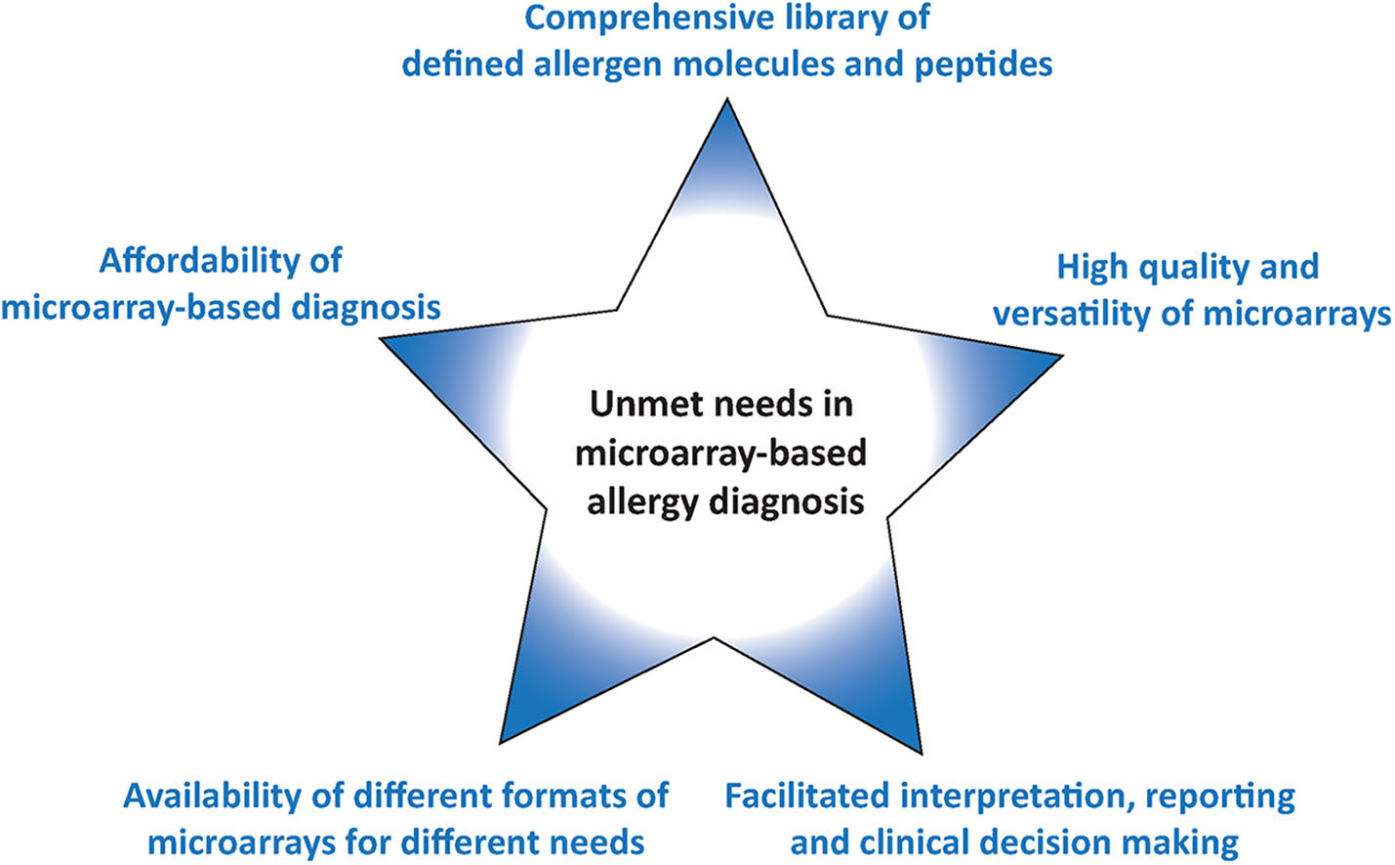
Unmet needs in microarray-based allergy diagnosis.

Since the first description of the use of microarrayed allergens in 2002 for allergy diagnosis the technology has become available world-wide and has been used extensively in research. However, several unmet needs remain which are summarized in **Figure 2** and discussed below.

### The Library of Allergen Molecules and Peptides

The allergen molecules and allergen derived peptides and their quality can be considered the heart of any allergen microarray because it determines what application can be addressed with the allergen chip. For example, it is important to cover the most common respiratory, food, venom, and other allergen sources by a representative collection of allergen molecules. In this context it should be noted that there are different opinions regarding the inclusion of certain allergen molecules in screening assays. For example, some argue that the inclusion of venom allergens may create ethical and legal issues because in case of a positive or negative test result one cannot predict or exclude a sting reaction. However, this is in principle applicable for every IgE test result which needs to be considered in the context of clinical information and/or results from provocation testing. Therefore others think that the inclusion of venom allergens in screening tests is not a problem provided the patients are adequately informed about the relevance of the IgE test results.

The addition of allergen-derived peptides may be interesting for example in the diagnosis of food allergy where sequential IgE epitopes play a role and for the monitoring of AIT-induced IgG responses. Since only small amounts of allergen are immobilized on microarrays it is important to use highly pure allergens of high quality to detect also low specific IgE responses. Natural allergen preparation containing impurities in terms of unrelated allergens or cross-reactive carbohydrates may give rise to unclear, false-positive test results. Although carbohydrates are highly cross-reactive, it seems that patients mount quite specific and selective IgE to certain carbohydrates which cannot be completely inhibited by pre-incubation of sera with a single carbohydrate (92) so that unclear background reactivity may remain. This is of particular relevance because IgE-reactive carbohydrates have been shown to have little or no clinical relevance. Accordingly it is important to establish a library of high quality allergen molecules and peptides which can be reproduced according to defined protocols. The allergen molecules should be preferentially made as recombinant, non-glycosylated allergens to avoid unclear results due to carbohydrate-specific IgE.

This allergen library should be as complete as possible to pick up every relevant IgE sensitization in a given population. For example it has been shown, that the MeDALL allergen chip, a customized allergen array based on the ImmunoCAP ISAC platform containing more than 170 allergen molecules (83), was more sensitive in picking up IgE sensitizations than traditional allergy tests based on comprehensive panels of allergen extracts for skin testing or IgE serology (93). In order to refine the panel of allergen molecules on a chip it will be necessary to investigate molecular IgE sensitization profiles in different populations in different countries and continents to define the allergen repertoire of a microarray suitable for allergy diagnosis in the whole world. Such a complete representation of allergen molecules seems very important because due to the high mobility of the world population allergen arrays representing only a local allergen repertoire will not be sufficient for diagnosis. The production of microarrays containing subsets of allergen molecules does not seem to have any advantages because the microarray technology does not set limitations regarding the numbers of molecules which can be immobilized, and the costs of goods for production are low due to the low amounts of allergen needed. However, one must consider that costs for quality control, validation, and registration of complex assays may increase costs considerably.

### New Materials May Increase the Quality and Versatility of Microarrays

There are basically two types of multiplex diagnostic platforms available. One contains allergen molecules adsorbed to microbeads, whereas the other platform is based on allergens which are immobilized on chips by micro-spotting. Microbead-based multiplex assay usually can accommodate only a limited number of less than 50 different allergen molecules in a single test and require quite expensive instruments such as Luminex readers or FACS-based technology for read out (94, 95). By contrast, microarrays allow measuring specific antibody reactivity to more than hundred different allergen molecules at the same time. There are currently two major types of allergen arrays available, the ImmunoCAP ISAC platform, using allergen molecules immobilized onto glass (Thermo Fisher ImmunoCAP ISAC Immuno-solid-phase Allergen Chip which contains 112 allergens from 51 allergen sources) (38) and an allergen macro-array prepared on the basis of a nitrocellulose membrane (*e.g.*, MADx Allergen Explorer ALEX; containing 282 allergens: 156 extracts and 126 components) (39). Both systems allow reasonable detection of allergen-specific IgE but one may consider increasing the quality of the arrays by selecting different materials for allergen immobilization. Already in the original patent application made for the ImmunoCAP ISAC technology, silicon-based surfaces have been considered as alternative to glass (96).

In fact, the high sensitivity of protein assays on microarray silicon slides has then been demonstrated (97, 98). The latter studies demonstrated optimized layers of thermally grown silicon oxide with highly reproducible thickness, low roughness, and fluorescence background which yielded fluorescence intensification due to the constructive interference between the incident and reflected waves of the fluorescence radiation. Furthermore, the studies suggested that by combining an optimized reflective substrate with a high performance surface chemistry may strongly improve the quality of diagnostic protein array by obtaining a 5–10 fold enhancement of the fluorescence signals when compared to glass surfaces. The favorable features of silicon slides have been further demonstrated for peptide arrays (99) and for the field of food allergy diagnosis (100).

Below, we have evaluated allergen microarrays based on silicon oxide surfaces and compared them with glass slides currently used in the ImmunoCAP ISAC platform confirming the higher sensitivity of the silicon technology. We are then also discussing advantages of this technology for the versatile production of different formats of allergen microarrays.

### Affordability of Microarray-Based Diagnosis

Currently available microarray-based allergy tests are relatively expensive although one has to consider that a single microarray test result provides more than hundred individual test results. For example costs for one array may range between 60 and 100 Euro depending on prices requested by different manufacturers in different countries, and additional costs for the processing of one sample may vary considerably 50–300 Euro depending on costs of laboratory facilities and personnel. Therefore, it will be necessary to decrease the costs of production for the micro-arrays and the costs for the test procedure which is currently performed by hand pipetting.

### Different Formats of Microarrays for Different Needs

As mentioned above currently available microarrays are made for manual operation and thus individual testing requires wet laboratory facilities and relatively expensive scanning equipment for the analysis of results. The available formats thus can be used for manual analysis of relatively limited numbers of samples and require trained personnel. Unfortunately, no automatization for the processing of the available allergen arrays is available which would allow large scale and fully automated analysis of large numbers of patients. Therefore, there is still an unmet need for different formats of allergen arrays allowing different applications such as the occasional analysis of few or single serum samples yielding fast results with a minimum of equipment, the medium scale analysis of several serum samples and the fully automated analysis of large numbers of sera (**Figure 2**).

Furthermore, allergen arrays should allow the analysis not only of allergen-specific IgE but also of other immunoglobulin isotypes as well as the visualization of the competitive activity of allergen-specific IgG on IgE binding for the monitoring of AIT.

### Interpretation, Reporting and Clinical Decision Making

Since allergen microarrays deliver test results for more than 100 different allergen molecules it is important that doctors who see allergic patients and wish to correctly interpret the sometimes complex test results keep themselves updated by continuous medical education. The transition of allergy diagnosis from the use of allergen extracts to allergen molecules requires knowledge regarding the characteristics of the individual allergen molecules. Thus molecular allergy diagnosis may be compared a bit with the switching from previous old telephones to mobile phones which offer many different additional applications that need to be explored by the user. The challenges of interpreting allergen microarray results may be met by machine learning approaches and other diagnostic algorithms in addition to continuous medical education (10, 101).

## A Comparison of Different Surfaces for Microarrays: Glass *Versus* Silicon

**Figure 3:**
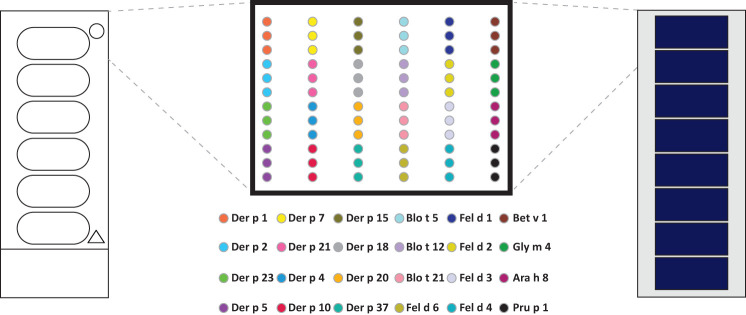
Outlay of prototype allergen microarrays made by printing on glass slides and assembled silicon elements. Order of house dust mite, mite, cat, and PR10 allergens microarrayed in triplicates on glass slides (left) and precut and assembled silicon chips-derived elements.

**Figure 4:**
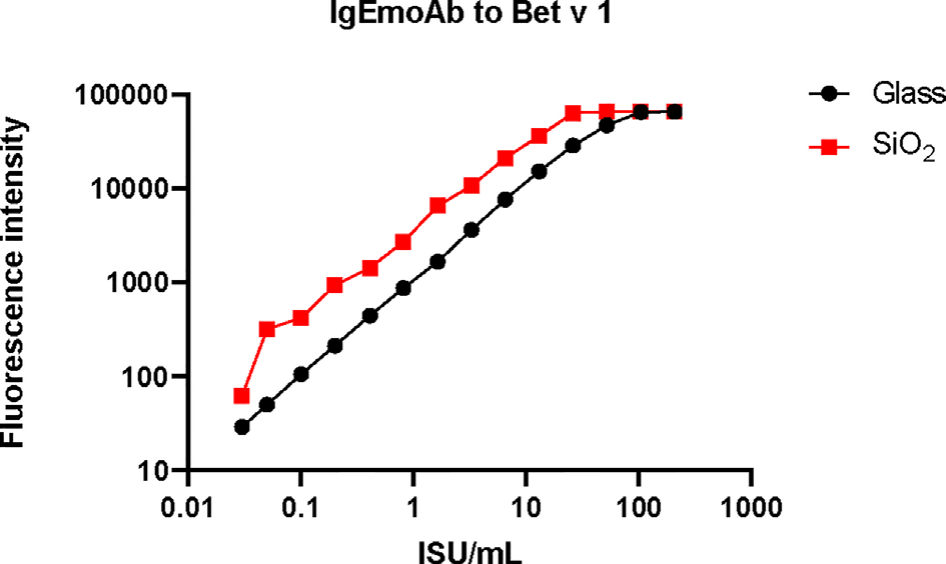
Sensitivity of the reactivity of a human monoclonal Bet v 1-specific IgE antibody to Bet v 1 printed on glass *versus* silicon. Shown are the fluorescence light intensities (y-axis) corresponding to different concentrations (x-axis) of the monoclonal human Bet v 1-specific IgE antibody (IgEmoAb).

In order to compare the glass surface which currently is used for ImmunoCAP ISAC with silicon slides (97–100) a panel of important respiratory allergens was spotted on the two surfaces and allergic patients’ IgE and IgG reactivity was assessed. A set of 24 allergens containing mite allergens (Der p 1, Der p 2, Der p 4, Der p 5, Der p 7, Der p 10, Der p 15, Der p 18, Der p 20, Der p 21, Der p 23, Der p 37, Blo t 5, Blo t 12 and Blot 21), cat allergens (Fel d 1, Fel d 2, Fel d 3, Fel d 4 and Fel d 6), and PR10 allergens (Bet v 1, Gly m 4, Ara h 8 and Pru p 4) were spotted in triplicates on glass and silicon wafers in the order described (**Figure 3**) (*Supplementary Materials and Methods*). In the first experiment we determined the sensitivity of IgE reactivity to Bet v 1 immobilized to glass and silicon chips using a human monoclonal chimeric IgE antibody (IgEmoAb) (102) (**Figure 4**). A two-fold serial dilution of IgEmoAb corresponding to 208–0.025 ISU/ml was used to detect Bet v 1. Silicon microarrays showed a five-fold higher fluorescence intensity of IgE reactivity of IgEmoAb in the range of 52–0.025 ISU/ml to Bet v 1 than the glass surface (**Figure 4**). The silicon surface allowed measuring the Bet v 1-specific IgE down to 0.025 ISU/ml which is much lower than the cut-off used in currently available microarray tests (0.3 ISU/ml) and the detection limit 0.1 ISU/ml used for certain research purposes.

**Figure 5:**
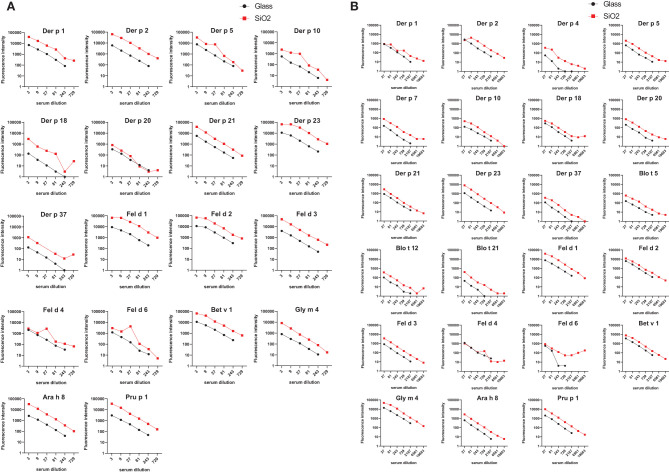
Comparison of IgE and IgG reactivity to HDM/mite, cat, and PR10 allergens which had been microarrayed on glass and silicon. A serum pool containing IgE and IgG antibodies against each of the tested allergens was tested for **(A)** IgE and **(B)** IgG reactivity to the individual allergens in different dilutions (x-axes). Fluorescence intensities corresponding to bound antibodies are shown on the y-axes.

**Figure 5** shows the comparison of IgE and IgG reactivity to allergens spotted on glass and silicon microarrayed chips determined with a serum pool from allergic patients with IgE reactivity against the tested allergen panel that was diluted 3- to 729-fold for IgE detection (**Figure 5A**) and 27- to 19,683-fold for IgG detection (**Figure 5B**). **Figure 5A** shows that the silicon surface yielded an approximately 10-fold higher IgE binding according to fluorescence intensity to all but one (*i.e.*, Der p 20) allergen compared to the glass surface. **Figure 5B** demonstrates that silicon was superior to glass also regarding IgG detection showing approximately five-fold higher IgG signals, and specific binding was detectable even at a dilution of 1:19,683 of the serum pool on silicon microarrays.

**Figure 6:**
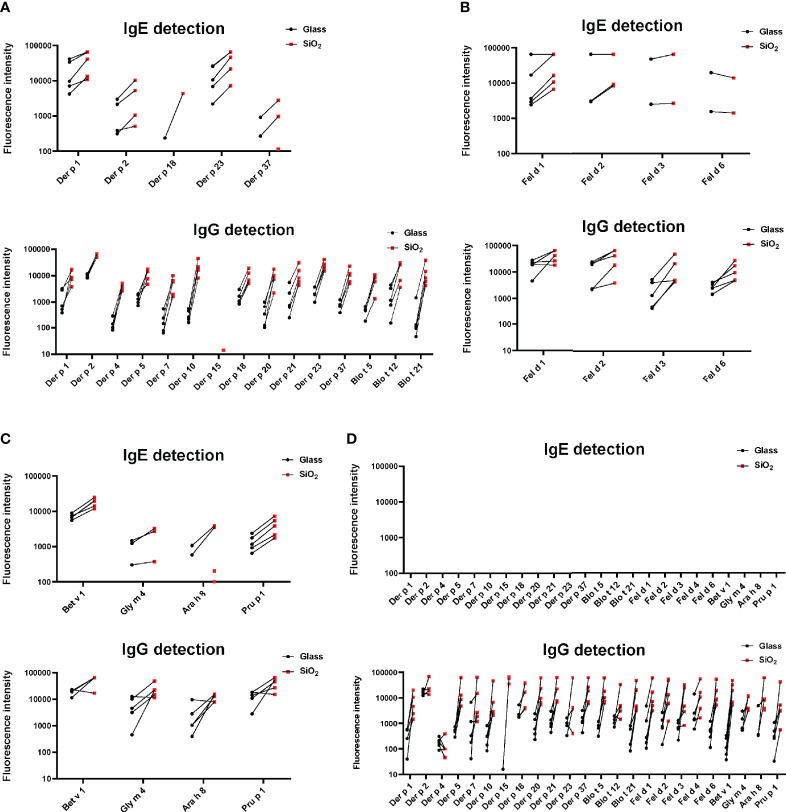
IgE- and IgG-reactivity (y-axes: fluorescence intensities corresponding to bound antibodies) of allergic patients to **(A)** microarrayed HDM/mite allergens, **(B)** cat allergens and **(C)** PR10 allergens (x-axes) and of non-allergic subjects to all tested allergens **(D)**. Glass: black dots; Silicon: red dots.

**Figure 7:**
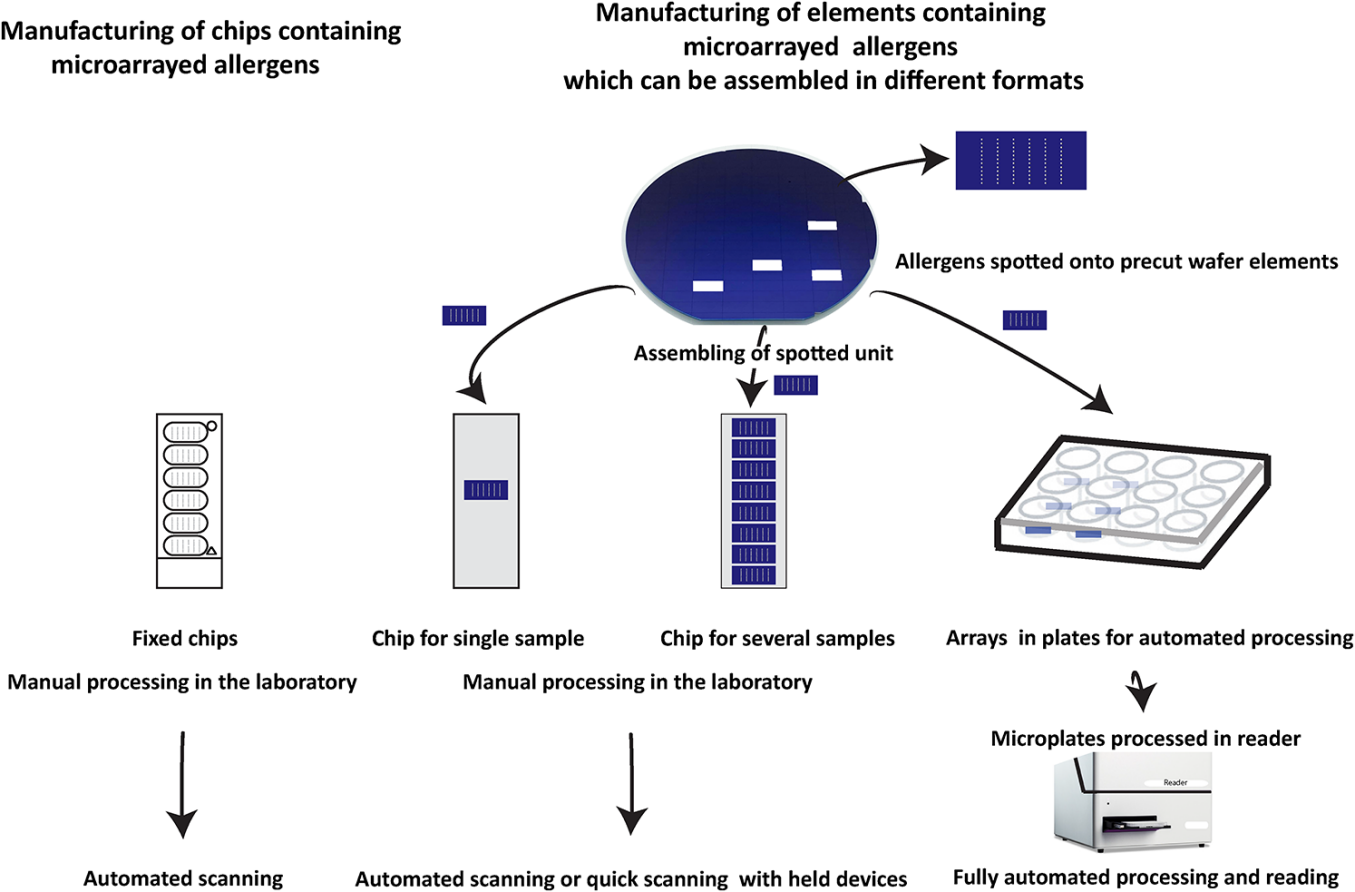
Different assembly of silicon-based microarray elements generates different microarray formats for different diagnostic needs. Currently available allergen chips represent a single format which cannot adapt to different needs as shown for ImmunoCAP ISAC. By contrast, silicon-based microarray elements can be assembled in different formats. For example, chips containing only one array for individualized rapid testing can be produced which due to the high sensitivity can be read in simple scanners or by using mobile phone-based cameras. Alternatively, chips containing up to eight microarrays can be assembled which allow manual testing of medium scale numbers of sera and subsequent analysis by inexpensive automated scanners. For large scale automated testing of large numbers of sera, silicon elements can be mounted in ELISA-type plates and subjected to automated testing in a closed ELISA-based instrument containing an incubation, washing and detection unit followed by a scanning unit.

Next, we tested sera from HDM and mite- (**Figure 6A**), cat- (**Figure 6B**), birch pollen allergic patients (**Figure 6C**) and non-allergic subjects (**Figure 6D**) for IgE and IgG reactivity to allergens microarrayed on silicon and glass. This experiment confirmed for almost all tested sera and allergens that allergens immobilized on silicon have higher IgE and IgG detection signals. Importantly, IgE detection was highly specific for glass and silicon because none of the non-allergic subjects showed detectable allergen-specific IgE reactivity (**Figure 6D**). Our results thus indicate that allergen microarrays based on silicon are superior to glass for IgE and IgG detection of allergens. It should be also noted that another advantage of silicon arrays is that spotting can be performed on small, precut silicon elements which can be assembled in different formats (**Figure 7**, right upper part).

## Manufacturing of MicroArray Elements and Subsequent Assembly Meets Different Needs in Allergy Diagnosis

In **Figure 7** we try to provide an overview of how microarrays based on silicon elements may contribute to innovation in allergy testing as compared to currently available chips and arrays used for IgE serology. Currently available allergen arrays are manufactured in one predetermined format which is a chip, containing one or several identically prepared allergen arrays (**Figure 7**, left part) which then need to be processed in the laboratory by hand pipetting.

Disadvantages of chips containing more than one array are that the spotting of the microarrays is performed directly on chips which are relatively large in comparison to a single silicon element. However, the single silicon elements can be arranged in much shorter distance close to each other for the spotting than preformed chips. Accordingly the spotting machine (microarray printer) makes much shorter movements when spotting closely assembled silicon elements as compared to premade chips which should speed up the production by shortening the production time. Another disadvantage of chips containing several microarrays is that one array of bad quality will lead to the discarding of a complete chip although the other arrays may meet the quality criteria. By contrast, when single silicon elements are used only the few poor quality elements will be discarded keeping the loss of material low. However, the most important advantage of microarrays based on single silicon elements is that after spotting, the single elements can be assembled in different formats for different uses. This allows producing chips containing only one microarray for fast testing of single serum samples. Furthermore, chips containing several silicon elements for testing of several sera can be assembled. Importantly, silicon elements can be also assembled in plates (*e.g.*, ELISA plate format) for automated processing of samples which can be incubated with samples, washed, developed, and read in machines without requiring hand pipetting. Thus microarrays printed on silicon elements allow assembling of different devices for testing based on one standardized element. Furthermore, silicon surfaces give 5–10-fold higher sensitivity as compared to glass which should allow detecting also low allergen-specific IgE levels with high precision, and the measurement can be done with very simple and inexpensive detection devices.

## Conclusion

Since the first description of allergen microarrays for allergy diagnosis almost 20 years ago, these multi-allergen tests have been successfully used to answer many research questions and have proved highly valuable for allergy diagnosis in multiple applications. However, several needs for improvement have remained unmet until today limiting the broad application of microarray-aided allergy diagnosis. We introduce here a novel concept for improving allergen microarray technology by showing that microarrays prepared on silicon offer higher sensitivity for the detection of specific IgE than the currently used glass surfaces and other surfaces with similar sensitivity as glass. Instead of spotting allergen arrays on preformed, inflexible devices we propose manufacturing of flexible silicon elements containing microarrays which then can be assembled in different formats. This allows addressing the different needs of allergy diagnosis ranging from manual testing of single or few sera to fully automated processing of large numbers of sera. Microarrays based on silicon elements are versatile arrays that can be easily produced. Using this technology it should be possible to decrease the costs of microarray testing to make the technology affordable to the health care systems. The heart of the allergen array is a library of high-quality allergen molecules. Microarrays utilizing low amounts of immobilized allergens allowing visualizing the interplay of allergen-specific IgE and IgG mimicking the *in vivo* situation and thus should deliver serological test results resembling the clinical sensitivity. Our vision for microarray-aided allergy diagnosis is to make available to the doctor the complete IgE reactivity profile of the patient already at the initial visit or during teleconsultation to achieve a complete diagnosis and personalized treatment without need for multiple time-consuming visits for the benefit of the patient and to reduce the costs for health care in allergy.

## Author Contributions

RV and HJH wrote the manuscript. RV, HJH and TS designed the figures and tables. HJH, RC, OA, MC, KR, AnK, OE, EF, AL, KN, ESa, and TS performed experiments and/or contributed materials. RC, OA, MC, ESa, AnK, KR, AlK, OE, EF, AL, ESm, MK, KN, SV, TS, HJH and RV critically read and revised the manuscript. All authors contributed to the article and approved the submitted version.

## Funding

This review was supported by the Danube Allergy Research Cluster funded by the Country of Lower Austria, by the MCCA PhD program and grant No P29398 of the Austrian Science Fund (FWF), by the Russian Academic Excellence Project 5-100, by a Megagrant of the Government of the Russian Federation, grant No 14.W03.31.0024, and by grant from HVD Life Science, Vienna, Austria.

## Conflict of Interest

RV has received research grants from HVD Life Science, Vienna, Austria, and Viravaxx, Vienna, Austria, and serves as a consultant for Viravaxx.

The remaining authors declare that the research was conducted in the absence of any commercial or financial relationships that could be construed as a potential conflict of interest.

## Abbreviations

IgE, Immunoglobulin E; IgG, Immunoglobulin G; Ab, antibody; AIT, allergen-specific immunotherapy; Ig, immunoglobulin; RAST, radioallergosorbent test; GP, general practitioner; OAS, oral allergy syndrome; RV, rhinovirus; HDM, house dust mite; Der p, *Dermatophagoides pteronyssinus*; Blo t, *Blomia tropicalis*; Fel d, *Felis domesticus*; PR10, pathogen-related protein 10; Bet v, *Betula verrucosa*; Gly m, *Glycine max*; Ara h, *Arachis hypogaea*; Pru p, *Prunus persica*.
