# Supplementary material for: Microarray-Based Allergy Diagnosis: Quo Vadis?
Source: Front Immunol. 2021 Feb 12;11:594978. doi: 10.3389/fimmu.2020.594978 (PMC7928321; doi:10.3389/fimmu.2020.594978)
Supplement: Supplementary file 1 [file DataSheet_1.docx]

**Materials and Methods**

**Allergic patients and non-allergic control subjects**

HDM-sensitized patients, cat-sensitized patients and non-allergic individuals were recruited with approval of the Ethics Committee of the Medical University of Vienna (EK 1641/2014), Vienna, Austria. Bet v 1-sensitized patients were recruited at the National Institute of Immunology, Moscow, Russia as described (1). The demographic and clinical characterization of allergic subjects and non-allergic control individuals was performed as described (1). HDM-allergic patients showed IgE reactivity to *D. pteronyssinus* ≥5.0 kUA/l as determined by ImmunoCAP (d1 ImmunoCAP, [Thermo Fisher](http://www.thermofisher.com/) Scientific/Phadia, Uppsala, Sweden), allergen-specific IgE in cat-allergic patients to cat dander was ≥2.7 kUA/l (e1 ImmunoCAP) and Bet v 1-sensitized patients had ≥9.7 kUA/l (t215 ImmunoCAP). Non-allergic individuals had no allergen-related symptoms and did not show IgE reactivity to any of the allergen molecules present on the MeDALL allergen chip (2).

**Allergens**

Recombinant Der p 2, Der p 5, Der p 7, Der p 18, Der p 20, Der p 21, Der p 23, Fel d 1 were expressed and purified as described (3-6). Der p 4 (Genbank accession number, [AF144060](http://www.ncbi.nlm.nih.gov/entrez/query.fcgi?cmd=search&db=nucleotide&doptcmdl=genbank&term=AF144060)), Der p 10 (Y14906.1), Der p 14 ([AF373221](http://www.ncbi.nlm.nih.gov/entrez/query.fcgi?cmd=search&db=nucleotide&doptcmdl=genbank&term=AF373221)), Der p 15 ([DQ078740](http://www.ncbi.nlm.nih.gov/entrez/query.fcgi?cmd=search&db=nucleotide&doptcmdl=genbank&term=DQ078740)), Der p 37 ([MG520330](http://www.ncbi.nlm.nih.gov/nuccore/MG520330)), Blo t 5 ([U59102](http://www.ncbi.nlm.nih.gov/nuccore/U59102)), Blot 12 ([U27479](http://www.ncbi.nlm.nih.gov/nuccore/U27479)), Blo t 21 ([AY800348](http://www.ncbi.nlm.nih.gov/nuccore/AY800348) ), Gly m 4 (X60043), and Pru p 1 (DQ251187) were expressed in *E. coli* BL21 (DE3) using the pET-17b expression plasmid (Novagen, Merck KGaA, Darmstadt, Germany) as recombinant proteins containing a hexahistidine tag at the C-terminus whereas Fel d 3 ([AF238996](http://www.ncbi.nlm.nih.gov/nuccore/AF238996).1), Fel d 4 ([AY497902](http://www.ncbi.nlm.nih.gov/nuccore/AY497902).1), and Ara h 8 (AY328088) were expressed a in *E. coli* BL21 (DE3) using the pET-27b plasmid as recombinant proteins with a C-terminal hexahistidine tag (Novagen, Merck KGaA, Darmstadt, Germany). Recombinant allergens were purified by Nickel-affinity chromatography as recommended in the Quiagen expression manual (Quigen, Hilden, Germany).

Purified natural Der p 1 and 2 were purchased from Citeq (Groningen, The Netherlands), recombinant Bet v 1 from Biomay (Vienna, Austria) and natural Fel d 2 and natural Fel d 6 were purchased from Sigma (Vienna, Austria) and Rockland antibodies and assays (Gilbertsville, PA, USA), respectively.

**Preparation of slides, slide coating and allergen spotting**

Silicon chips of 8x17 mm with 90 nm silicon dioxide (SiO_2_) were purchased from Silicon Valley Microelectronics, Inc. (Santa Clara, CA, USA) and mounted in frames (Ing. Prägler Ges.m.b.H., Vienna, Austria). Silicon chips and glass slides (Paul Marienfeld GmbH & Co. KG, Lauda-Königshofen, Germany) were coated with MCP-2 (Lucidant Polymers, Sunnyvale, CA. USA) diluted 1:100 in 0.9 M (NH_4_)_2_SO_4_ water solution. After incubation of slides with MCP-2 in the dark for 15 min at room temperature, the slides were rinsed with distilled H_2_O and dried using a bench centrifuge (200 g, 2 min). Coated slides were stored under vacuum at 4°C. Allergens were spotted in triplicate on MCP-2-coated silicon and glass slides using a SciFlex array spotter (Scienion, Berlin, Germany) according to the allergen layout described in Fig. 2. Three hundred pL of each allergen (c=1 mg/ml) were spotted at a distance of 500 μm from each other. The pH of allergen samples was adjusted with 750 mM Na_2_HPO_4_ buffer to pH 8.4. After spotting, the slides were incubated overnight in the dark in a 75 % humidity chamber to immobilize the allergens. After overnight incubation, the chips were sprayed with blocking buffer (30 mmol/L ethanolamine in PBS containing 0.1 % Tween-20) and incubated for 30 min at room temperature. After 30 min incubation, the slides were immersed in liquid plate sealer (CANDOR Bioscience GmbH, Wangen, Germany) for 15 min. Slides were dried by centrifugation (200 g, 2 min) and stored under vacuum at 4°C until use.

**Bioassays**

Slides containing spotted allergens were immersed with wash buffer (PBS containing 1% Tween-20) and dried by centrifugation (200 g, 1 min) before serum application. Thirty microliter of undiluted (IgE detection) and diluted samples (serum and monoclonal IgE antibody) were incubated on microarray chips for 2 hours. For control purposes slides were incubated with sample diluent alone (Thermo Fisher Scientific/Phadia, Uppsala, Sweden). After incubation, the slides were washed with wash buffer and dried by centrigfugation (200 g, 1 min). Thirty microliter of a mouse monoclonal anti-human IgE antibody (Roche, Basel, [Switzerland](https://en.wikipedia.org/wiki/Switzerland)) and goat anti-human IgG antibody (Fab´)_2_ (Jackson ImmunoResearch, West Grove, PA, USA) (c= 1 mg/ml) conjugated with DyLight™ 550-2xPEG NHS Ester (Thermo Fisher Scientific, Waltham, Massachusetts, USA) were added and incubated in the dark for 30 min at room temperature. The conjugation of the detection antibodies to DyLight™ 550-2xPEG NHS Ester was performed according to the manufacturer' s instructions. Unbound antibodies were washed away with wash buffer and then with distilled H_2_O. Slides were dried by centrifugation (200 g, 2 min). Fluorescence signals were detected by a TECAN Power Scanner (Grödig, Austria) at 25% laser power and 50% photomultiplier (PMT) gain for IgE measurement and at 10% laser power and 10% PMT gain for IgG measurement. Fluorescence intensities were analyzed using Luxscan software (Beijing, China). The buffer control (i.e., result obtained only with buffer and detection antibodies) was subtracted from each of the result and median fluorescence intensity (FI) from triplicate data were analyzed. The cut-off values for IgE and IgG detection corresponding to 0.1 IU/mL were 100 and 10 fluorescence intensities (FIs) for IgE and IgG, respectively.

**References**

1. Elisyutina O, Lupinek C, Fedenko E, Litovkina A, Smolnikov E, Ilina N, et al. IgE-reactivity profiles to allergen molecules in Russian children with and without symptoms of allergy revealed by micro-array analysis. *Pediat Allerg Imm-Uk* (2020). doi: 10.1111/pai.13354. PubMed PMID: WOS:000574770800001.

2. Lupinek C, Wollmann E, Baar A, Banerjee S, Breiteneder H, Broecker BM, et al. Advances in allergen-microarray technology for diagnosis and monitoring of allergy: the MeDALL allergen-chip. *Methods* (2014) 66(1):106-19. Epub 2013/10/29. doi: 10.1016/j.ymeth.2013.10.008. PubMed PMID: 24161540; PubMed Central PMCID: PMCPMC4687054.

3. Huang HJ, Curin M, Banerjee S, Chen KW, Garmatiuk T, Resch-Marat Y, et al. A hypoallergenic peptide mix containing T cell epitopes of the clinically relevant house dust mite allergens. *Allergy* (2019) 74(12):2461-78. Epub 2019/06/23. doi: 10.1111/all.13956. PubMed PMID: 31228873; PubMed Central PMCID: PMCPMC7078969.

4. Resch Y, Blatt K, Malkus U, Fercher C, Swoboda I, Focke-Tejkl M, et al. Molecular, structural and immunological characterization of Der p 18, a chitinase-like house dust mite allergen. *PLoS One* (2016) 11(8):e0160641. Epub 2016/08/23. doi: 10.1371/journal.pone.0160641. PubMed PMID: 27548813; PubMed Central PMCID: PMCPMC4993390

5. Sarzsinszky E, Lupinek C, Vrtala S, Huang H, Hofer G, Keller W, et al. Expression in Escherichia coli and purification of folded rDer p 20, the arginine kinase from Dermatophagoides pteronyssinus: A possible biomarker for allergic asthma. *Allergy Asthma Immunol Res*. In press.

6. Gronlund H, Bergman T, Sandstrom K, Alvelius G, Reininger R, Verdino P, et al. Formation of disulfide bonds and homodimers of the major cat allergen Fel d 1 equivalent to the natural allergen by expression in Escherichia coli. *J Biol Chem* (2003) 278(41):40144-51. Epub 2003/05/07. doi: 10.1074/jbc.M301416200. PubMed PMID: 12732623.
